# Association Between MMR Status and Prognostic Pathological Factors in Endometrioid Endometrial Cancer—A Single-Center Retrospective Study

**DOI:** 10.3390/cancers17223605

**Published:** 2025-11-08

**Authors:** Cezary Miedziarek, Hubert Bochyński, Katarzyna Bociańska, Michał Potograbski, Piotr Tyburski, Mikołaj Piotr Zaborowski, Ewa Nowak-Markwitz

**Affiliations:** 1 Division of Gynaecological Oncology, Department of Gynaecology, Poznan University of Medical Sciences, 61-701 Poznań, Poland; 2 Doctoral School, Poznan University of Medical Sciences, 61-701 Poznań, Poland; 3 Institute of Bioorganic Chemistry, Polish Academy of Sciences, 61-772 Poznań, Poland

**Keywords:** endometrial cancer, mismatch repair deficiency, LVSI, microsatellite instability, prognostic factors

## Abstract

**Simple Summary:**

Clinical and pathological heterogeneity of endometrial carcinoma is not fully investigated, particularly in relation to mismatch repair (MMR) status. In practice, tumor profiles combining dMMR with adverse features may support therapy escalation, whereas favorable features support de-escalation. Identifying dMMR tumors has therapeutic relevance, guiding the consideration of immune checkpoint inhibitors. Previous studies have reported various findings regarding the impact of MMR deficiency, especially with respect to clinical progression of the disease, tumor grade, and the presence of adverse histopathological features such as lymphovascular space invasion (LVSI). By investigating these associations in our retrospective patient cohort, we aim to clarify the impact of MMR status on disease aggressiveness and to contribute evidence that may guide clinical decision-making. This study is centered on histopathological risk factors of endometrial cancer.

**Abstract:**

**Background/Objectives**: Prognostic assessment in endometrial cancer (EC) is based on clinical and pathological features such as histological type, FIGO stage, tumor grade, LVSI, P53 status, and hormone receptor expression. Recent molecular research has distinguished four EC subtypes, with MMR status (pMMR vs. dMMR) providing clinically relevant stratification due to its predictive value for immunotherapy. The present study aims to compare dMMR and pMMR tumors in terms of the prevalence of adverse histopathological prognostic factors. **Methods**: This retrospective study included 179 patients with endometrioid endometrial carcinoma (EEC) treated at the authors’ institution (1 January 2023–31 August 2025). Patients were classified by MMR status (pMMR vs. dMMR) based on immunohistochemistry, and clinicopathological variables, including FIGO stage, myometrial invasion depth, tumor grade, LVSI, ER/PR expression, and P53 status, were analyzed. Normality was assessed using the Shapiro–Wilk test. Categorical variables were tested with chi-square or Fisher’s exact tests, reporting odds ratios with 95% CI, while continuous variables were compared using the Mann–Whitney test and presented as median (IQR) with the Hodges–Lehmann difference and 95% CI. Multivariable logistic regression with Wald tests was performed. **Results**: dMMR tumors accounted for 29.05% of all cases. Patients in the dMMR group were significantly more likely to present with FIGO stage III/IV disease (*p* = 0.036) and to exhibit LVSI (*p* = 0.008). No differences were observed between the groups with respect to tumor grade, estrogen receptor positivity, progesterone receptor positivity, or the prevalence of deep myometrial invasion. The most frequent pattern of protein loss in the dMMR population was concurrent loss of MLH1 and PMS2. **Conclusions**: In the studied population, dMMR tumors more frequently exhibited adverse prognostic features of EC, such as advanced stage of disease and lymphovascular space invasion. This suggests the potential for effective immunotherapy in this patient group.

## 1. Introduction

Endometrial cancer (EC) is a malignancy with a favorable prognosis. Considering all patients, the 5-year survival rate for this cancer is approximately 81% [1]. Prognostic assessment in EC depends on multiple clinical and pathological factors. The most important prognostic factors include histological tumor type, clinical stage of the disease, tumor grade, presence of lymphovascular space invasion (LVSI), and the presence of *P53* gene mutations. Estrogen receptors (ER) and progesterone receptors (PR) positivity generally tracks with favorable biology and disease course, whereas receptor loss often coexists with adverse features [2,3].

The outcome of recent research on EC has been the identification of molecular subtypes of the disease, with practical implications. Based on The Cancer Genome Atlas (TCGA) analysis, EC has been molecularly classified into four subtypes: POLE ultramutated, microsatellite instability-high (MSI-H), copy-number low (CN-L), and copy-number high (CN-H) [4]. Accordingly, major guidelines incorporate molecular subtype into postoperative risk stratification and adjuvant therapy decisions (e.g., potential de-escalation for *POLE* ultramutated and escalation for *TP53*-mutated) [5]. Microsatellites are short repeats composed of a few nucleotides that are distributed throughout the genome. During DNA replication, errors can occur within these repeats. In normal cells, such errors are corrected by the DNA mismatch repair (MMR) system. When MMR is intact (pMMR), microsatellite length is preserved. When MMR is deficient (dMMR), errors accumulate, leading to microsatellite instability (MSI). In EC, tumors are therefore commonly classified as pMMR or dMMR, a distinction with important clinical implications, as dMMR/MSI-high tumors are particularly responsive to immune checkpoint inhibition [6,7,8]. Current guidelines endorse universal tumor testing for MMR deficiency in endometrial cancer, typically by MMR immunohistochemistry (IHC) with MSI testing (PCR or NGS) as an alternative or confirmatory approach [9]. On IHC, loss of MSH2, MSH6, or isolated PMS2 strongly suggests Lynch syndrome (LS) and directs germline testing toward the corresponding MMR gene, whereas MLH1 may also suggest sporadic MLH promoter hypermethylation (MLH1-PHM) with transcriptional silencing. Rare LS-like cases due to constitutional MLH1 methylation have been reported, but with limited need for genetic counseling, because they are not associated with heritable (germline) alterations [10]. Evidence linking MMR status to histopathologic aggressiveness in EC, including FIGO stage, LVSI, grade, and depth of invasion, remains inconsistent across studies. This knowledge gap motivates the comparison of pMMR and dMMR tumors using routinely reported pathologic features.

MMR status is a recognized predictor of response to immunotherapy in EC [6]. Consequently, scientific studies increasingly consider dMMR and pMMR populations as two distinct entities, requiring separate investigations due to their differences in treatment response. We hypothesized that dMMR tumors exhibit more aggressive pathological features, as they entail high mutational burden and a dysregulated immune microenvironment, alongside genomic instability [1,2,5]. The aim of the present study is to compare dMMR and pMMR EC patients with respect to the prevalence of pathological prognostic factors.

## 2. Materials and Methods

**Figure 1:**
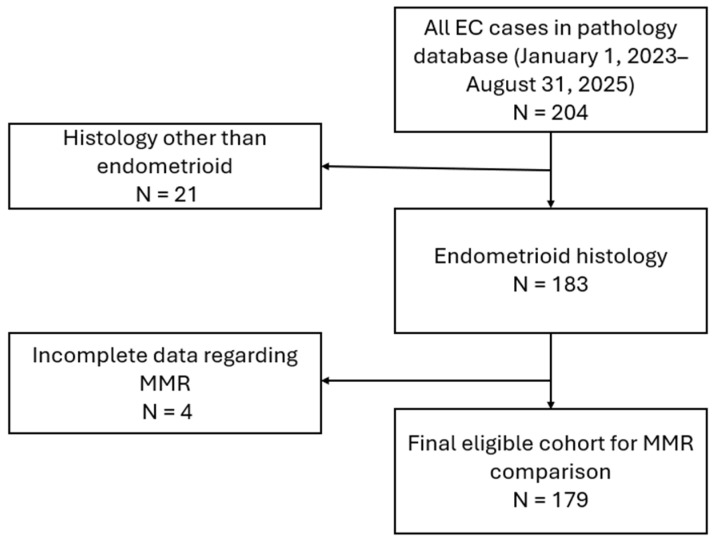
Flow-chart presenting the selection of final cohort for MMR comparison.

This retrospective study included patients treated for EC over a two-year period (1 January 2023–31 August 2025) at the authors’ affiliated institution. We enrolled patients consecutively, applying prespecified exclusions (non-endometrioid histology, missing core variables). The study was conducted at the Department of Gynecologic Oncology, Poznan University of Medical Sciences. The Bioethics Committee of Poznan University of Medical Sciences confirmed that, due to the retrospective nature of the study, no additional approval was required (Confirmation KB-655/25). The study was conducted in accordance with the principles outlined in the Declaration of Helsinki. Details of patient selection are presented in the flow chart (Figure 1).

The study included patients who were diagnosed with EC. Among these, only those with an endometrioid histopathological subtype (EEC) were analyzed. Histopathological diagnoses based on biopsy and postoperative specimens, surgical descriptions, and imaging studies were reviewed. Patients were divided into two main groups according to their MMR status, determined by immunohistochemical testing for the loss of expression of the MLH1, PMS2, MSH2, and MSH6 proteins: pMMR (proficient MMR) and dMMR (deficient MMR). Focal MMR loss was considered noninterpretable. We relied on unequivocally stained regions and repeated IHC as needed. Reproducible focal loss suggestive of clonal deficiency was flagged as suspicious for dMMR, prompted genetic testing [11], and revealed MSI-high in all such cases (n = 8). Patient data regarding tumor stage according to FIGO were collected, and for analysis purposes, patients were categorized into two groups: those with stage I and II disease and those with stage III and IV disease. Independently, patients with deep (≥50%) and superficial (<50%) myometrial invasion were identified. Tumors were also classified by grade into low-grade (G1 and G2) and high-grade (G3). LVSI status was considered positive if it was substantial or focal, and negative if it was absent. ER and PR status were defined according to the Allred score, where scores 0–2 were considered negative and scores 3–8 were considered positive [12]. Wild-type P53 (P53-wt) was defined as heterogeneous or variable nuclear staining with an internal control present. Aberrant (P53-abn) was defined by diffuse strong nuclear overexpression, complete absence of nuclear staining (null) with intact internal controls, or cytoplasmic staining with absent nuclear staining [13,14]. Mutation analysis of the *POLE* and *TP53* genes was not performed using molecular methods, as these tests were not yet implemented in routine practice at the whole time of data collection. Additionally, in our center, immunohistochemical results for P53 generally correlate well with genetic mutation analysis. Antibodies used for ICH are presented in Table 1.

MSI assays were not available for all dMMR cases, particularly those from the early data collection period. However, in the tested cases (n = 34), all tumors were found to be MSI-high. Non-standard immunohistochemical patterns (e.g., isolated loss of a single MMR protein) prompted expedited referral for genetic counseling and evaluation for Lynch syndrome, as per institutional practice. These data are not included in the present study, which focuses on histopathologic determinants. It has to be noted that, as MSI assays were not available for all tumors and MMR status was determined by IHC, this approach can misclassify a minority of cases, e.g., those with non-standard immunohistochemical patterns.

Statistical analysis, including both descriptive and comparative methods, was conducted using Microsoft Excel and RStudio 2024.12.1. Group comparisons for categorical variables used chi-square or Fisher’s exact tests, as appropriate. For 2 × 2 tables, we additionally reported odds ratios (OR) with 95% confidence intervals calculated by Fisher’s exact method. Continuous variables were compared using the Mann–Whitney test and are summarized as median (IQR) with Hodges–Lehmann differences and 95% CI. Observations with missing data were excluded from bivariate analyses. We performed multivariable analyses for FIGO and LVSI (multivariable logistic regression with Wald tests). For the FIGO analysis, we excluded depth of myometrial invasion and LVSI, as these are elements of the staging definition. ER and PR status were also excluded due to substantial missing data and a significantly different proportion of missing values between the dMMR and pMMR groups (*p* = 0.0287, chi-square). For the LVSI analysis, we excluded FIGO (the FIGO classification incorporates LVSI) and ER and PR status due to the high proportion of missing data. We prespecified covariates and did not use stepwise selection. Complete case analysis, ORs with 95% CIs, and AUC are reported.

Missing data were handled by complete-case analysis for all inferential tests and models. ‘Unknown’ values were used only for descriptive summaries. We prespecified this approach to avoid ad hoc imputation and because of high rate of missingness for ER/PR data.

## 3. Results

The study group consists of 179 female patients (mean age 63.76 ± 10.43) with EC of the endometrioid histological subtype treated at our clinic. Selected epidemiologic factors—age, body weight and BMI—are summarized in Table 2. A total of 127 (70.95%) were classified as pMMR and 52 (29.05%) as dMMR. A detailed comparison of the two groups with respect to the analyzed parameters is presented in Table 3. The category “Unknown” is included for descriptive purposes only. For the analysis, we employed complete-case analysis, excluding observations with missing values in any variable included in a given model.

In bivariate tests, patients in the dMMR group were significantly more likely to be LVSI-positive compared with the pMMR group. Patients with dMMR tumors more frequently presented with FIGO stage III–IV disease. The groups did not differ significantly in histological grade, P53 status, estrogen receptor positivity, progesterone receptor positivity, and the prevalence of deep myometrial invasion. The groups were also comparable in terms of age, body weight, and BMI.

In a multivariable model (outcome: dMMR vs. pMMR) including FIGO, LVSI, grade, P53, and myoinvasion, FIGO III/IV was not significant: aOR = [1.95] (95% CI [0.72–5.25], *p* = 0.187). LVSI remained significant: aOR = [2.96] (95% CI [1.25–7.04], *p* = 0.014). It has to be noted, that the model showed modest discrimination (AUC = [0.646], 95% CI [0.546–0.735]) (Table 4).

In multivariate analysis, the presence of LVSI was independently associated with dMMR status (aOR [3.38], 95% CI [1.46–7.81]; *p* = 0.004). Additionally, it was associated with deep myometrial invasion (aOR [12.45], 95% CI [5.15–30.11]; *p* = <0.001, model discrimination: AUC = [0.807] (95% CI [0.743–0.867]). Advanced stage (FIGO III/IV) was independently associated with dMMR status (aOR [2.68], 95% CI [1.11–6.47]; *p* = 0.028). Additionally, it was associated with high histological grade (aOR [3.85], 95% CI [1.45–10.19]; *p* = [0.007]). Model discrimination: AUC = [0.709] (95% CI [0.604–0.811]) (Table 5 and Table 6).

An additional analysis was performed to evaluate the combinations of mismatch repair protein loss associated with dMMR. The most common pattern of absent expression on immunohistochemistry was combined loss of MLH1 and PMS2, which occurred in 78.8% of cases (Table 7).

## 4. Discussion

The dMMR population accounted for 29.05% of patients, which is consistent with previous reports indicating that this alteration occurs in approximately 25–30% of ECs [15,16]. It is worth noting that these analyses encompass all histopathological subtypes of EC. Nevertheless, the endometrioid subtype remains the most prevalent.

Data regarding the impact of MMR status on the clinical and pathological characteristics of EC remain inconclusive [17]. In our studied population, patients with EC and dMMR status were significantly more likely to present with a higher FIGO clinical stage and exhibited LVSI more frequently. It has to be noted that our results are associations and do not imply causation. Several studies have demonstrated an association between MMR status and the clinical stage of EC at diagnosis, where dMMR endometrial carcinomas are considered to exhibit a more aggressive clinical course [18]. In these studies, patients with dMMR EC are considered more likely to present with FIGO stage III or IV disease [19,20,21]. Moreover, they tend to have larger tumor size and a higher incidence of lymph node involvement, both of which are associated with advanced FIGO classification [19]. Reports also indicate a significantly lower prevalence of FIGO stage I disease among dMMR EC compared with pMMR tumors, with a relative shift toward stages II–IV [22]. On the other hand, several large studies have suggested that dMMR EC is more often present at lower tumor stages compared with MMR-proficient cases. Nonetheless, these reports consistently highlight that the dMMR subgroup more frequently exhibits metastatic parameters such as LVSI [17,23,24]. Given that dMMR cancers are more often associated with adverse clinicopathologic features, the implementation of immunotherapy is particularly valuable, especially in patients with advanced disease, as this subgroup derives the most significant benefit from immune checkpoint inhibition [18,25,26]. It is worth noting that differences in FIGO stage across studies may reflect heterogeneity in patient selection and IHC protocols, as well as variations in reporting schemes (e.g., stage I reported separately vs. pooling I + II as ‘early’ and III + IV as ‘advanced’). Differences in ethnic composition across referenced studies and the monoethnic nature of our cohort are also relevant to between-population comparisons.

LVSI is defined as the presence of malignant cells within lymphatic and/or vascular spaces. It represents an adverse prognostic factor in EC, being associated with lymph node metastasis, an increased risk of pelvic recurrence, and reduced overall survival. The impact is significant for both 3-year and 5-year survival rates, with the latter decreasing by 13–25% in LVSI-positive disease [27]. Women with dMMR tumors were more likely to have higher-grade cancers and more frequent LVSI [28], the latter consistent with our results.

Available analyses provide conflicting evidence regarding the association between tumor grade and MMR status. Some studies have demonstrated that high-grade tumors occur more frequently in the dMMR population, whereas others report them to be less common or similarly distributed, as observed in our cohort [28,29,30]. This inconsistency highlights the need for additional population-based studies in this area, particularly since tumor grade continues to be a key factor influencing therapeutic decision-making in early-stage disease [31].

Studies emphasize that in the majority of EEC, including dMMR tumors, estrogen and progesterone receptors are expressed, and P53 displays a wild-type pattern [32,33]. This is consistent with the findings of our analysis. Positive expression of estrogen and progesterone receptors, as well as the absence of *TP53* mutations, represent favorable prognostic factors [34,35]. High-grade tumors constitute approximately 15–20% of endometrial cancers when a binary grading scheme is used. They are associated with worse clinical outcomes and more frequent adverse molecular alterations, including *TP53* mutations [2]. The relationship between grade and the prevalence of ER/PR positivity remains controversial and varies across studies [34].

Additional analysis of immunohistochemistry patterns in dMMR EC cells reveals that the most prevalent phenotype is the loss of expression of both MLH1 and PMS2 proteins, accounting for approximately 70–80% of cases, which is in agreement with the outcomes from our own cohort. This reflects the instability of the PMS2 protein in the absence of its MLH1 partner [36,37]. The MSH2 and MSH6 co-loss is the second most common combination (15–20%), typically indicative of MSH2 mutations [26]. Less frequently, isolated MSH6 or PMS2 losses occur (5–10%), corresponding to mutations in the respective genes [38,39]. Interestingly, in our patient population, the second most common finding was isolated loss of single proteins, namely MSH6, MLH1, or MSH2, not MSH2 and MSH6 co-loss, which is considered a less common situation [27].

## 5. Limitations

A limitation of our study is the lack of genetic testing for *POLE* and *TP53* mutations. The presence of POLE mutation represents an independent favorable prognostic factor in patients with endometrial carcinoma [40]. Genetic testing for *TP53* mutations was also not included, as genetic assays were introduced into clinical practice after the initiation of data collection for this study. Furthermore, immunohistochemistry has been shown to correlate well with genetic testing for *TP53* [41,42,43]. Therefore, it is unlikely that the omission of these analyses would have significantly affected our results.

However, in clinical practice, without *POLE* and *TP53* sequencing, residual TCGA misclassification is possible. Favorable *POLE*-mutant and poor-prognosis *TP53*-aberrant tumors may be grouped solely based on clinical and pathological factors. While this analysis relied on IHC surrogates to show histologic features, full molecular profiling, including *POLE* and *TP53* genetic testing, will be undertaken for any patient for whom it is expected to change clinical decision-making. This is important because *POLE*-ultramutated tumors (even with adverse histology such as LVSI or deep invasion) generally have an excellent prognosis and may warrant de-escalation of adjuvant therapy, whereas *TP53*-mutated tumors carry an unfavorable prognosis and often justify treatment escalation.

Although MMR IHC generally aligns well with MSI-high status in endometrial cancer, numerous studies have documented discordant cases. Therefore, the absence of MSI testing in every case should be regarded as a limitation.

As a single-center study, our cohort may reflect referral bias due to the case mix specific to our institution. It is limited by sample size, which may reduce precision and the generalizability of estimates. The benefit of a single-center design is histopathological consistency and uniform IHC.

## 6. Conclusions

EECs differ in their clinical and histopathological phenotypes depending on MMR status. Our findings are in line with reports indicating that dMMR tumors are characterized by a more aggressive clinical course and a higher prevalence of adverse histopathological features. In our cohort, this was reflected by a greater frequency of advanced FIGO stage and more common occurrence of LVSI in the dMMR group. These observations underscore the importance of adjuvant treatment, particularly immunotherapy, which has proven to be especially effective in this subset of patients. Future multicenter studies should integrate MMR status with comprehensive molecular profiling (*POLE* and *TP53*) to refine risk stratification and guide adjuvant therapy. Clinically, dMMR is a validated predictive biomarker for response to immune checkpoint inhibition, while LVSI is an established adverse prognostic feature. Together, these variables could support risk-adapted enrichment in immunotherapy trials, e.g., prioritizing dMMR tumors and considering dMMR with LVSI as a high-risk disease. Such strategies may increase event rates and therapeutic signal detection while aligning with routine pathology workflows. Prospective, biomarker-driven trials with standardized staging and pathology review are needed to confirm their benefits.

## Figures and Tables

**Table 1:** Antibodies used for ICH. All immunohistochemical stains were performed on the DAKO Autostainer Link 48 using the EnVision™ FLEX, High pH detection system; all antibodies were supplied by DAKO.

Target	Antibody	Clone
P53	Flex RTU Monoclonal Mouse Anti-Human, P53 Protein	DO-7
ER	Flex RTU Monoclonal Rabbit Anti-Human, Estrogen Receptor alfa	EP1
PR	Flex RTU Monoclonal Mouse Anti-Human, Progesterone Receptor	PgR 636
MLH1	Flex RTU Monoclonal Mouse Anti-Human, MutL Protein Homolog 1, MLH1	ES05
PMS2	Flex RTU Monoclonal Rabbit Anti-Human, Postmeiotic Segregation Increased 2, PMS2	EP51
MSH2	Flex RTU Monoclonal Mouse Anti-Human, MutS Protein Homolog 2, MSH2	FE11
MSH6	Flex RTU Monoclonal Rabbit Anti-Human, MutS Protein Homolog 6, MSH6	EP49

**Table 2:** Selected epidemiologic factors summary. HL—Hodges–Lehmann differences; CI—confidence interval.

Variable	Total (n = 179)	pMMR (n = 127)	dMMR (n = 52)	*p*-Value	HL	95% CI
Age [years] mean ± SD	63.76 ± 10.43	63.31 ± 10.21	64.85 ± 10.97	0.255	2.00	−1.00–5.00
Body weight [kg] median, IQR	77 (67–93)	77 (67–93)	76.5 (68–92)	0.709	1.00	−7.00–4.00
BMI [kg/m^2^] median, IQR	29.67 (25.71–34.85)	29.62 (25.86–34.29)	30.18 (25.06–35.3)	0.868	−0.19	−2.55–1.89

**Table 3:** Study group characteristics. The designation ‘unknown’ in the myometrial invasion section refers to cases where the available material was insufficient for assessment. P53 and hormone receptor status were not determined in specimens with only superficial myometrial infiltration and limited tissue. OR—odds ratio; CI—confidence interval.

Variable	Total (n = 179)	pMMR (n = 127)	dMMR (n = 52)	*p*-Value	OR	95% CI
FIGO				0.036 *	**2.63**	**1.04–6.63**
- I/II	150 (83.80%)	111 (87.40%)	39 (75.00%)			
- III/IV	27 (15.08%)	14 (11.02%)	13 (25.00%)			
- Unknown	2 (1.12%)	2 (1.57%)	0 (0.00%)			
Grade				0.422	1.57	0.58–4.04
- Low (G1/G2)	148 (82.68%)	106 (83.46%)	42 (80.77%)			
- High (G3)	26 (14.35%)	16 (12.60%)	10 (19.23%)			
- Unknown	5 (2.79%)	5 (3.94%)	0 (0.00%)			
LVSI				0.008 *	**2.52**	**1.24–5.21**
- Positive	77 (43.02%)	46 (36.22%)	31 (59.62%)			
- Negative	100 (55.87%)	79 (62.20%)	21 (40.38%)			
- Unknown	2 (1.12%)	2 (1.57%)	0 (0.00%)			
Estrogen receptors				0.765	2.25	0.24–109.25
- Positive	135 (75.42%)	93 (73.23%)	42 (80.77%)			
- Negative	6 (3.35%)	5 (3.94%)	1 (1.92%)			
- Unknown	38 (21.23%)	29 (22.83%)	9 (17.31%)			
Progesterone receptors				0.352	3.71	0.47–169.42
- Positive	132 (73.74%)	90 (70.87%)	42 (80.77%)			
- Negative	9 (5.03%)	8 (6.30%)	1 (1.92%)			
- Unknown	38 (21.23%)	29 (22.83%)	9 (17.31%)			
P53				0.335	0.50	0.11–1.64
- Aberrant	22 (12.29%)	18 (14.17%)	4 (7.69%)			
- Wild-type	156 (87.15%)	108 (85.04%)	48 (92.31%)			
- Unknown	1 (0.56%)	1 (0.79%)	0 (0.00%)			
Myometrial invasion				0.902	1.10	0.54–2.28
- ≥50%	104 (58.10%)	73 (57.48%)	31 (59.62%)			
- <50%)	72 (40.22%)	52 (40.94%)	20 (38.46%)			
- Unknown	3 (1.68%)	2 (1.57%)	1 (1.92%)			

* To highlight statistically significant values.

**Table 4:** Multivariable logistic regression with Wald tests—outcome: dMMR vs. pMMR. AUC = [0.646], 95% CI [0.546–0.735]. Complete cases = 169; events (dMMR) = 51. OR—odds ratio; CI—confidence interval.

Variable	OR	95% CI	*p*-Value
FIGO (III/IV)	1.95	0.72–5.25	0.187
Grade (high)	1.26	0.48–3.28	0.642
LVSI (present)	2.96	1.25–7.04	0.014 *
P53 (aberrant)	0.88	0.25–3.03	0.838
Myometrial invasion (deep)	0.50	0.21–1.21	0.123

* To highlight statistically significant values.

**Table 5:** Multivariable logistic regression with Wald tests—outcome: LVSI present vs. absent. AUC = [0.807], 95% CI [0.743–0.867]. Complete cases = 169; events (LVSI present) = 72. OR—odds ratio; CI—confidence interval.

Variable	OR	95% CI	*p*-Value
MMR (dMMR)	3.38	1.46–7.81	0.004 *
Grade (high)	1.74	0.64–4.75	0.281
P53 (aberrant)	0.53	0.16–1.72	0.289
Myometrial invasion (deep)	12.45	5.15–30.11	<0.001 *

* To highlight statistically significant values.

**Table 6:** Multivariable logistic regression with Wald tests—outcome: FIGO I/II vs. III/IV. AUC = [0.709], 95% CI [0.604–0.811]. Complete cases = 173; events (FIGO III/IV) = 26. OR—odds ratio; CI—confidence interval.

Variable	OR	95% CI	*p*-Value
MMR (dMMR)	2.68	1.11–6.47	0.028 *
Grade (high)	3.85	1.45–10.19	0.007 *
P53 (aberrant)	1.37	0.35–5.37	0.654

* To highlight statistically significant values.

**Table 7:** Combinations of loss of expression of MMR-related proteins.

Pattern of MMR Protein Loss	Number of Cases (Percent)	Pattern of MMR Protein Loss	Number of Cases (Percent)
MLH1, PMS2	41 (78.8%)	MLH1, PMS2, MSH6	1 (1.9%)
MSH6	4 (7.7%)	MSH2	1 (1.9%)
MLH1	2 (3.8%)	PMS2, MSH2, MSH6	1 (1.9%)
MSH2, MSH6	2 (3.8%)		

## Data Availability

Data are contained within the article.
